# Immunomodulatory Effect after Irreversible Electroporation in Patients with Locally Advanced Pancreatic Cancer

**DOI:** 10.1155/2019/9346017

**Published:** 2019-05-12

**Authors:** Chaobin He, Jun Wang, Shuxin Sun, Yu Zhang, Shengping Li

**Affiliations:** ^1^Department of Hepatobiliary and Pancreatic Surgery, State Key Laboratory of Oncology in South China, Collaborative Innovation Center for Cancer Medicine, Sun Yat-sen University Cancer Center, Guangzhou 510060, China; ^2^Department of Ultrasonics, State Key Laboratory of Oncology in South China, Collaborative Innovation Center for Cancer Medicine, Sun Yat-sen University Cancer Center, Guangzhou 510060, China; ^3^State Key Laboratory of Ophthalmology, Zhongshan Ophthalmic Center, Sun Yat-sen University, Guangzhou, Guangdong 510060, China

## Abstract

**Purpose:**

Irreversible electroporation (IRE) has been demonstrated to be a safe and effective method for locally advanced pancreatic cancer (LAPC). The aim of this study was to evaluate the immunomodulatory effect after IRE and to evaluate the prognostic value of variations of the immune parameters in LAPC patients after IRE.

**Methods:**

Peripheral blood samples of 34 patients were obtained preoperatively and on the third day (D3) and seventh day (D7) after IRE, respectively. The phenotypes of lymphocytes were analyzed by flow cytometry, and dynamic changes of serum levels of cytokines, complement, and immunoglobulin were assayed by enzyme-linked immunosorbent assay. Receiver operating characteristic (ROC) curve and concordance index (C-index) were used to compare the survival predictive ability.

**Results:**

There was a transitory decrease followed by a steady increase for CD4^+^ T cell, CD8^+^ T cell, NK cell, IL-2, C3, C4, and IgG while a reverse trend was detected for Treg cell, IL-6, and IL10 after IRE. The alteration of CD8^+^ T cell between D3 and D7 was identified as a prognostic factor for both overall survival (OS) and progression-free survival (PFS). The values of ROC curve (AUC) and C-indexes of the alteration of CD8^+^ T cell for OS and PFS were 0.816 and 0.773 and 0.816 and 0.639, respectively, which were larger than those of other immune or inflammation-based indexes.

**Conclusions:**

This study presented the first evidence of IRE-based immunomodulatory in patients with LAPC. The alteration of CD8^+^ T cell between D3 and D7 showed relatively good performance and could be used as an effective tool for prognostic evaluation for LAPC patients after IRE.

## 1. Introduction

Pancreatic adenocarcinoma is a lethal disease with extremely poor prognosis, which also represented the seventh and sixth leading causes of cancer-related death in the world and in China, respectively. The 5-year survival rate is only 5% [1, 2]. Surgical resection is the only chance to obtain curative treatment while it is only suitable for less than 20% of patients with this disease [3]. Approximately 40% of new cases are diagnosed with locally advanced pancreatic cancer (LAPC), which is characterized by the involvement of major vascular structures, such as celiac trunk, superior mesenteric artery, leading to unresectable but nonmetastatic diseases [4]. Currently, the treatment for LAPC remains a huge challenge due to the poor prognoses of this disease. Limited responses and little impact on survival or life were achieved after the standard treatments, which was mainly systemic chemotherapy [5, 6]. Moreover, the high rates of adverse events due to the toxicity of chemotherapy limited the use and promotion of treatment, such as the combination chemotherapy of 5-fluorouracil, leucovorin, irinotecan, and oxaliplatin (FOLFIRINOX), even though it was shown to display some progression in improving the survival of patients with LAPC [7–9]. Therefore, it is necessary to evaluate new treatment to optimize common therapeutic approaches. Nowadays, local therapies were shown to improve the prognosis of LAPC patients with varying degrees of success [10].

Irreversible electroporation (IRE), a nonthermal ablation technique, is established as a local ablative therapy for patients with LAPC with promising outcomes of increasing overall survival (OS) from 12 months to 25 months [11]. It is a novel local destructive method based on the transmission of high voltage currents through the tumor via needles, leading to cell membrane defects and apoptotic death [12, 13]. Additionally, during the induction process of apoptotic death by IRE, the structure and composition of the tumor microenvironment are changed, inducing an intense inflammatory cell response, which is characterized by the infiltration of immune cells [14]. It was shown that this IRE-induced immunomodulatory was not only limited to the ablated areas, but also a systemic reaction [15]. Thus, IRE could be regarded as a potential immunomodulatory treatment and might induce extensive changes of immune cells or indexes after ablation.

So far, data is rare on the predictive factors of IRE outcome in patients with LAPC. For this novel and powerful treatment of LAPC, further prognostic markers are urgently needed to choose patients with relatively better prognosis. Moreover, early information of the efficacy of treatment during the first days after IRE would be highly appreciated as therapy may be intensified by other treatments, such as immune therapy, chemotherapy, and radiotherapy, while the regular evaluation of therapy by imaging is only done about 1 month after IRE treatment. For the candidates of predictive factors, circulating biochemical markers may be the promising ones, for their relationship with cancer disease, the immediate therapy effect, and the immunological response of the organism to treatment. More importantly, as failures were achieved for the immune-checkpoint therapies in pancreatic cancer due to the low rates of neoantigen expression and mutation events [16], exploring the alterations and evaluating the prognostic effect of immune cells and indexes might open the prospect of using immune-checkpoint therapies in patients with LAPC.

Here, immunomodulatory effect of IRE was examined by analyzing alterations of several immune cells and indexes in patients with LAPC. We aimed to evaluate the response to IRE therapy during the early treatment phase and identify their role in prognosis.

## 2. Materials and Methods

### 2.1. Patients

This study was retrospectively designed. Consecutive patients who were newly diagnosed with LAPC at Sun Yat-sen University Cancer Center between August 2015 and August 2017 were included in this study. The inclusion criteria were as follows: (1) pathologically confirmed pancreatic adenocarcinoma and radiologically confirmed LAPC. LAPC was defined per the seventh edition of the AJCC staging system for pancreatic cancer, which describes LAPC as arterial encasement of either the celiac axis or superior mesenteric artery or unreconstructable superior mesenteric or portal vein involvement, with no evidence of metastatic disease from abdominal and thoracic computed tomography [17, 18]; (2) IRE therapy as the initial treatment. A total of 11 patients were excluded based on the following exclusion criteria: (1) other treatments, including surgical resection and RFA before IRE (seven patients); (2) existing metastatic implants before IRE (one patient); (3) heart arrhythmia and a history of second primary malignant tumors (one patient); (4) missing information of parameters or lost to follow-up (two patients). This study was approved by the Institutional Review Board of Sun Yat-sen University Cancer Center. All procedures performed in present study involving human participants were in accordance with the ethical standards of institutional and/or national research committees and the 1964 Helsinki Declaration and its later amendments or similar ethical standards. Written informed consent was obtained from patients prior to treatment.

### 2.2. Clinical Data Collection

The following clinical and radiological data were retrieved from medical record archived at Sun Yat-sen University Cancer Center, including age, gender, tumor size, tumor grade, tumor site, white blood cell (WBC) count, platelet (PLT) count, serum levels of alanine transaminase (ALT), aspartate aminotransferase (AST), alkaline phosphatase (ALP), glutamyl transpeptidase (GGT), albumin (ALB), total bilirubin (TBIL), indirect bilirubin (IBIL), C-reactive protein (CRP), carcinoembryonic antigen (CEA), and carbohydrate antigen 19-9 (CA19-9). The inflammation-based indexes, including neutrophil-to-lymphocyte ratio (NLR), platelet-to-lymphocyte ratio (PLR), prognostic index (PI), and modified Glasgow Prognostic Score (mGPS), were also entered into this study. The thresholds for the clinical or radiological variables were used as the cutoff values. With the cutoff value of 1.47 and 165.29, NLR and PLR were associated with the optimal Youden indexes for OS and progression-free survival (PFS) prediction, respectively. The defined score of other inflammation-based indexes, such as PI and mGPS, had been described in previous studies [19].

### 2.3. Treatment Procedure

The NanoKnife IRE equipment from Angiodynamics System (Queensbury, NY, USA) was used. General anesthesia with deep neuromuscular block was adopted. To create an electric field around the tumor, 3 to 6 probes were used according to the size and location of the tumor. Ultrasound was used to guide the placement of all probes, and adequate space between probes was then confirmed. The generator unit software was used to analyze the probe configuration data of the ultrasound and provided optimal voltage and pulse length delivery. If the tumor size was larger than 1.5 cm in the axial plane, a pull-back technique with the same procedure was performed to cover the entire area of ablation.

### 2.4. Sample Collection

All blood samples were collected before the hypothesis of this study was known. The blood samples were collected using Na-heparin plasma tubes from enrolled patients before IRE (preOP) and then on days 3 (D3) and 7 (D7) after IRE. Isolation of peripheral blood mononuclear cells (PBMCs) was processed immediately using Hypaque-Ficoll (Promega) and frozen in liquid nitrogen in 5% (v/v) plus 95% (v/v) autologous serum [20].

### 2.5. Flow Cytometry Analysis

Frozen PBMCs were thawed in a 37°C water bath and then cultured overnight at 37°C in RPMI-1640 (Gibco BRL) supplemented with 5% human AB type serum and labeled with FITC-, APC-, and/or PE-conjugated murine anti-human monoclonal antibodies. The CD3, CD3CD4, CD3CD8, CD3CD16CD56, and CD4CD25 phenotype of lymphocytes were sequentially analyzed by flow cytometry (FACS caliber, 4 color system, BD Bioscience, CA, US).

### 2.6. Assays of Immune Parameters

The quantitative sandwich enzyme immunoassay technique (ELISA kit, R&D system, Minneapolis, MN) was adopted to measure serum concentrations of cytokines, including IL-2, IL6, IL-10, interferon-*γ* (IFN-*γ*), and tumor-necrosis factor (TNF). During the procedure of measure, 50 to 100 *μ*l of assay diluent was added to the 96-well polystyrene microplate, which was precoated with murine monoclonal antibody against IL-2, IL-6, IL-10, IFN-*γ*, and TNF. Serum samples were incubated at 37°C for 2 hours and then the plates were aspired and washed three times. Same incubation was repeated after 200 micoliters of conjugate was added. Then, plates were incubated at 37°C for 20 to 30 minutes after 200 *μ*l of substrate solution was added. Finally, 50 *μ*l of stop solution was added to the plates. A microplate reader (ClinicalBio 128c, Austria) was used to read optimal density (OD) within 30 minutes at 450 nm wavelength, whose references were set to 550 and 620 nm.

A Beckman ARRAY 360 System (Beckman Coulter, Galway, Ireland) was used to evaluate the concentrations of several humoral immune parameters, including C3, C4, IgA, IgM, and IgG. Specific antibodies were measured by enzyme-linked immunosorbent assay (ELISA). After the incubation, the microplate reader (ClinicalBio 128c, Austria) was used to read OD within 30 minutes at 450 nm wavelength, whose reference was set to 630 nm [20].

### 2.7. Follow-Up

The follow-up procedure was performed in accordance with previous publications and recommendations [21, 22]. OS was defined as the duration from treatment until death or the last follow-up. PFS was defined as the duration from treatment until the date when disease progression was diagnosed or until the last follow-up. The last follow-up was completed on September 30, 2018.

### 2.8. Statistical Analysis

Continuous variables were compared using an independent sample t-test and the Mann-Whitney U test. Binary categorical variables were compared using the chi-square test. OS and PFS curves were analyzed using the Kaplan-Meier method, and differences between the groups were identified using the log-rank test. Univariate analysis was performed to assess the significance of parameters. Multivariate analysis was performed using the Cox regression model for the variables that were found to be significant in the univariate analysis, and the corresponding 95% confidence intervals (CIs) were calculated. ROC curves and C-indexes were used to compare the survival predictive ability. Two-tailed P values < 0.05 were considered statistically significant. All statistical analyses were performed using the R statistical package (R software version 3.4.2; R Foundation for Statistical Computing, Vienna, Austria).

## 3. Results

### 3.1. Patient Characteristics

In the present study, a total of 34 patients with LAPC were retrospectively included in this study. All patients have received IRE therapy. There were 18 (52.9%) female patients and 16 (47.1%) male patients. The median age was 59.5 years (range 45-73 years). Patient characteristics were summarized in Table 1. Large size and moderate differentiation were the most commonly seen features of tumors. Most patients had lower values of inflammatory indexes, such as PLR, PI, and mGPS, while patients with higher values of NLR occupied the majority of all patients. For the whole study cohort, there were only 4 patients whose TBIL was higher than 100 umol/L. Complications after IRE treatment in patients with LAPC were also evaluated (Table 2). The most frequently reported complications were pain (3 of 34 patients) and hypotension (3 of 34 patients).

### 3.2. Modulation of Circulating Immune Cells

**Figure 1:**
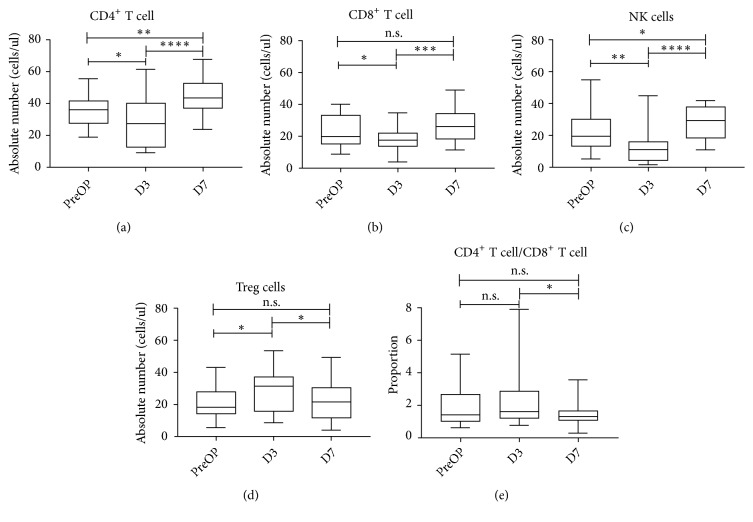
Distribution of serum concentration of CD4+ T cell (a), CD 8+ T cell (b), NK cell (c), Treg cell (d), and the ratio of CD4+ T cell/CD8+ T cell (e) before, 3 days, and 7 days after IRE therapy, indicating medians, interquartile range, 5th and 95th percentiles, and extreme values. ^*∗*^p < 0.05; ^*∗∗*^p < 0.01; ^*∗∗∗*^p < 0.001; ^*∗∗∗∗*^p < 0.0001. NK cell: natural kill cell; Treg cell: regulatory T cell; IRE: irreversible electroporation.

To investigate how IRE influences circulating immune cells, these cells were phenotypically characterized by evaluating the absolute number of helper T cell (CD4^+^ T cell, identified as CD3^+^CD4^+^), cytotoxic T cell (CD8^+^ T cell, identified as CD3^+^CD8^+^), regulatory T cell (Treg, identified as CD4^+^CD25^+^FoxP3^+^), and natural killer cell (NK cell, identified as CD3^−^CD16^+^CD56^+^) before (preOP) and after IRE treatment (D3 and D7). It was shown that the absolute numbers of CD4^+^ T cell (*p*<0.05), CD8^+^ T cell (*p*<0.05), and NK cell (*p*<0.01) were decreased immediately after IRE (D3), followed by a steady increase in the next few days (D7) (*p*<0.001). However, the trend for Treg cell reversed between preOP and D7 (*p*<0.05). The NK cell showed the most dramatic inverse effect for each time interval. Huge alterations of CD4^+^ T cell and CD8^+^ T cell were observed while there was a significant decrease in the ratio of CD4^+^ T cell to CD8^+^ T cell from D3 to D7 (*p*<0.05) (Figure 1).

### 3.3. Modulation of Circulating Cytokines and Humoral Immune Parameters

**Figure 2:**
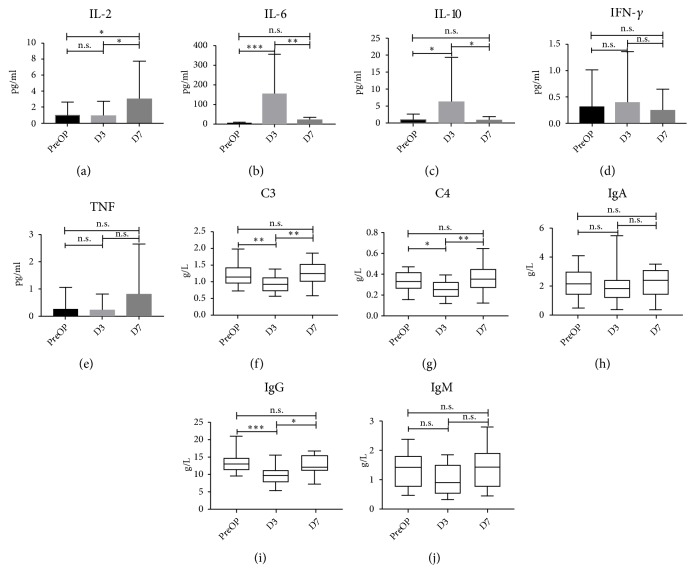
Distribution of serum concentration of IL-2 (a), IL-6 (b), IL-10 (c), IFN-*γ*(d), TNF (e), C3 (f), C4 (g), IgA (h), IgG (i), IgM (j) before, 3 days, and 7 days after IRE therapy. ^*∗*^p < 0.05; ^*∗∗*^p < 0.01; ^*∗∗∗*^p < 0.001. IL: interleukin; IFN-*γ*: interferon-*γ*; TNF: tumor-necrosis factor; C3: complement 3; C4: complement 4; IgA: immunoglobulin A; IgG: immunoglobulin G; IgM: immunoglobulin M; IRE: irreversible electroporation.

For a more complete understanding of the IRE-associated alteration of immune, analyses of the plasma concentration of several cytokines were conducted. Marked changes were observed for interleukin-2 (IL-2) (*p*<0.05), IL-6 (*p*<0.001), and IL-10 (*p*<0.01). IRE dramatically increased circulating IL-6 and IL-10 at D3 but these decreased at D7 (all* p*<0.05). Although no changes of IL-2 at D3 were observed, there was a significant increase from D3 to D7 (*p*<0.05). On the contrary, IRE did not significantly alter plasma concentration of IFN-*γ* and TNF (*p*>0.05). Moreover, we analyzed the plasma concentration of several general humoral immune parameters (complement: C3 and C4; immunoglobulin: IgA, IgG, and IgM). C3, C4, and IgG notably decreased immediately after IRE (D3) (all* p*<0.05) but significantly increased within one week (all* p*<0.01). There were no significant changes for concentration of IgA and IgM (all* p*>0.05) (Figure 2).

### 3.4. Comparison of Survival Stratified by Changes of Immune Cells and Parameters

**Figure 3:**
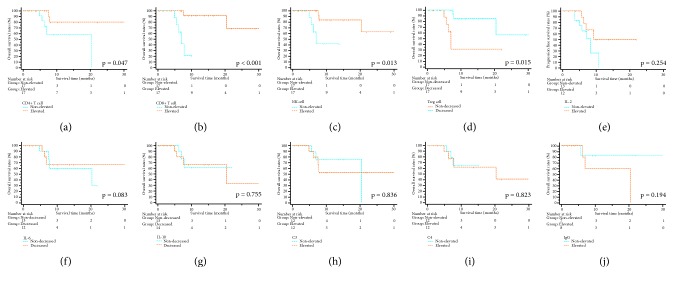
The survival curves of overall survival stratified by immune cells and parameters. Alteration of CD4+ T cell (a), CD 8+ T cell (b), NK cell (c), Treg cell (d), IL-2 (e), IL-6 (f), IL-10 (g), C3 (h), C4 (i), and IgG (j). NK cell: natural kill cell; Treg cell: regulatory T cell; IL: interleukin; C3: complement 3; C4: complement 4; IgG: immunoglobulin G; IRE: irreversible electroporation.

**Figure 4:**
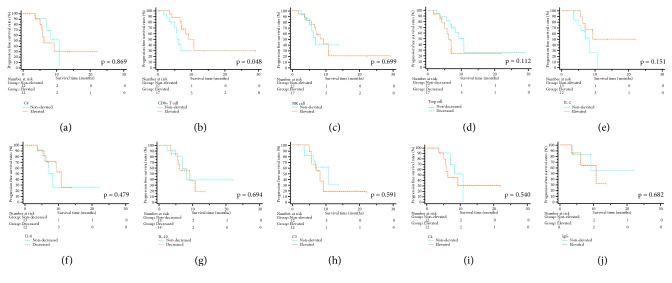
The survival curves of progression-free survival stratified by immune cells and parameters. Alteration of CD4+ T cell (a), CD 8+ T cell (b), NK cell (c), Treg cell (d), IL-2 (e), IL-6 (f), IL-10 (g), C3 (h), C4 (i), and IgG (j). NK cell: natural kill cell; Treg cell: regulatory T cell; IL: interleukin; C3: complement 3; C4: complement 4; IgG: immunoglobulin G; IRE: irreversible electroporation.

In the whole study cohort, there were 27 (79.4%) patients alive at the end of follow-up. The cumulative 1-year and 2-year OS rates were 69.9% and 52.4%, respectively. To evaluate the prognostic value of immune cells and parameters, the elevated or decreased group of these variables was defined by the threshold, which was the median value of the alterations between D3 and D7. In the subgroup analyses for OS, patients with an increase of CD4^+^ T cell (*p*=0.047), CD8^+^ T cell (*p*<0.001), and NK cell (*p*=0.013) or a decrease of Treg cell (*p*=0.015) had significant better OS than others. There were no significant differences with regard to OS when it was stratified by changes of cytokines, including IL-2, IL-6, and IL-10 (all* p>0.05)*. In addition to these variables, alteration of C3, C4, and IgG did not lead to significant differences in OS (all* p>0.05)* (Figure 3). Regarding PFS, significant survival benefit could be obtained from an increase of CD8^+^ T cell (*p*=0.048) while the alterations of other immune cells or parameters were not significantly associated with PFS (Figure 4).

### 3.5. Univariate and Multivariate Analyses of OS and PFS

In Cox regression analysis, the increase of CD8^+^ T cell was associated with increased OS and PFS [Elevated vs Nonelevated, OS, HR=0.039, 95%CI, 0.002-0.780,* p* = 0.034; PFS, HR=0.418, 95%CI, 0.138-0.954,* p*=0.049] in all patients. Moreover, there were no other prognostic factors for OS and the remaining one prognostic factor for PFS was NLR (> 1.47 vs ≤ 1.47, HR=3.425, 95%CI, 1.002-12.616,* p*=0.046) (Table 3).

### 3.6. Comparison of Predictive Value of the Immune Cells and Inflammation-Based Indexes

**Figure 5:**
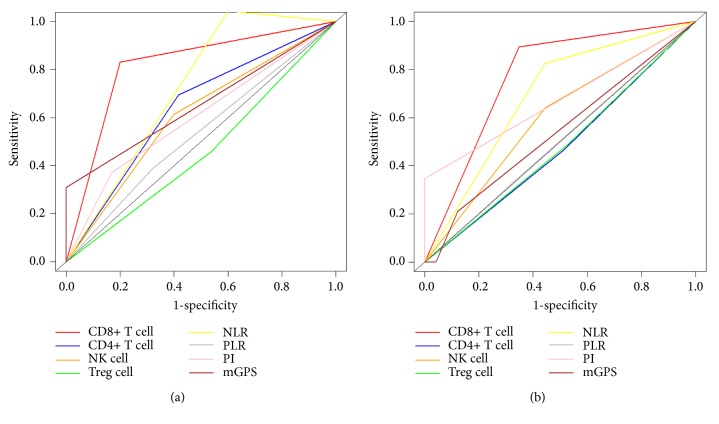
Comparison of ROC curves of alteration of immune cells, cytokines, or inflammation-based indexes, for predicting OS (a) and PFS (b) in patients with LAPC after IRE therapy. ROC: receiver operating characteristic; OS: overall survival; PFS: progression-free survival; LAPC: locally advanced pancreatic cancer; IRE: irreversible electroporation.

ROC curves were used to compare the sensitivity and specificity of survival prediction among the immune cells, parameters and inflammation-based indexes (Figure 5). The values of AUC of alteration of CD8^+^ T cell for OS and PFS prediction were 0.816 and 0.773, respectively, which were both higher than those of other immune parameters or inflammation-based indexes (Table 4). In terms of comparisons of C-indexes for OS prediction, the value of alteration of CD8^+^ T cell was 0.816 (95%CI 0.711-0.921), which was higher than that of other factors. In terms of PFS prediction, CD8^+^ T cell also displayed relatively high value of 0.639 (95%CI 0.523-0.755), showing significant better predictive power (Table 5).

## 4. Discussion

In this study, an immunomodulatory effect was demonstrated by altering lymphocytes, cytokines, and humoral immune parameters in patients with LAPC after IRE. It was the first evidence for IRE-based immune modulation in LAPC patients. It was shown that there was a transitory decrease followed by a steady increase for CD4^+^ T cell, CD8^+^ T cell, NK cell, IL-2, C3, C4, and IgG while a reverse trend was observed for Treg cell, IL-6, and IL10 after IRE. Other circulating cytokines, including TNF and IFN, were also evaluated. In terms of IFN, IFN-*γ* plays the most important role in its immunostimulatory and immunomodulatory effects, compared with the ability to inhibit viral replication directly, which is the main function of IFN-*α* [23, 24] or IFN-ß [25]. Therefore, IFN-*γ* and TNF were analyzed while they both failed to show obvious alteration. In addition, the alteration of CD8^+^ T cell between D3 and D7 was identified as prognostic factor for OS and PFS and first showed both a convenient and effective prognostic value in patients with LAPC after IRE. When compared with the traditional inflammation-based scores, the alteration of CD8^+^ T cell exhibited a better predictive value for both OS and PFS.

For LAPC patients after ablation therapy, several studies have revealed the changes of individual counts of T cell and subset ratios [26–28]. Alessandro G et al. compared the concentration of CD4^+^ and CD8^+^ T cell before and after radiofrequency ablation (RFA) and revealed an increase of above-mentioned T cells from the third day after treatment [26]. In the study conducted by Ketevan M et al., a more significant decrease in the expression of CD4^+^CD39^+^ T cell was observed after RFA, compared with operation [27]. In animal study, IRE therapy, which induced an increase tumor infiltration of CD3^+^ cells, was reported to be more effective in immunocompetent tumor than in immunocompromised tumors [28]. Furthermore, Martin et al. reported that IRE induced an obvious decrease in the absolute number of Treg cell in patients with LAPC [29]. Similar to Martin's study, our study showed a transitory increase followed by a remarkable decrease for Treg cell, along with a steady increase of effective T cells and humoral immune parameters after IRE. As an inflammation-inducing treatment, IRE not only directly destroys tumor cells, but also results in a release of tumor-associated neoantigens, which may stimulate the cellular and humoral immune of the body. Then, the numbers of immunocytes and production of humoral immune parameters will increase due to the potentiation of cellular and humoral immune. Moreover, it was shown that heat-shock proteins released from the destroyed tumor cells had an adjuvant effect and acted as an alarm for antitumor T cell-mediated immunity [30]. Therefore, IRE may be a mean of significant effort to overcome the immunosuppressive “cold” tumor microenvironment in LAPC and a potential treatment window of opportunity for immune-check-point therapy was suggested by increasing the effector T cells and decreasing immunosuppressive Treg cells. In addition, for these patients, prior biliary drainage procedure or a hepaticojejunostomy during open procedure was performed. Also, no serious complications, such as abdominal infection and pancreatic fistula, were observed in all patients after IRE therapy. Therefore, the influence of hyperbilirubinemia or infection after IRE on the alteration of immune cells was minimized. Although detained changes of immune cells had been described in patients with LAPC after IRE, the clinical performance of these changes in survival prediction was still unclear, thereby limiting their value.

In the next step of the present study, we evaluated the prognostic factors for OS and PFS and showed that elevation of CD8^+^ T cell was associated with favourable OS and PFS in LAPC patients after IRE. This can be explained by a stimulated host immune response which might limit the progression and invasion of tumor, and therefore, better survival was achieved. This can be proved by previous studies in which strong relationships were observed between immune toxicity and metastasis [31]. Metastases were more frequently observed in patients with lower density of immune effector cells [32], which was in accordance with our results. Furthermore, the predictive power of the alteration of several immune cells and inflammation-based indexes were compared in this study. It was demonstrated that the alteration of CD8^+^ T cell was superior to other indexes. In addition, as a robust and economic method, the alteration of CD8^+^ T cell can be obtained from peripheral blood sample fast and easily and can be used widely in clinical practice. Although there was a correlation between immune cells and inflammation-based indexes [27], the alteration of CD8+ T cell could still provide additional prognostic value in patients with the same levels of inflammation-associated situation. Maybe they can be considered as complements for predicting the prognosis of LAPC patients after IRE. However, a slightly lower value of AUC for CD8+ T cell in PFS prediction suggested that, compared with PFS, maybe OS was affected more greatly by the changes of immune system. Different from tumor-infiltrating CD8^+^ T cells, which were shown to play more important role in determine local progression, compared with prognosis [33, 34]. LAPC is a systemic disease other a local disease. In the present study, peripheral blood samples were collected before and after IRE treatment and were analyzed by flow cytometry for CD8^+^ T cells. Therefore, it was thought that the human immune system played a more important role in long-term survival than in local control. Similar to the present study, in the study conducted by Chen et al. [35], it was shown that alteration of CD8^+^ T cells was the only independent prognostic factor for OS, other than PFS. This may partly explain the different impact on survival from the alteration of CD8^+^ T cells. However, this difference needed to be further explored by further studies.

The comparison of AUC and C-indexes of the alteration of immune cells or inflammation-based indexes was conducted. Although the alteration of CD8^+^ T cell exhibited the most significant effects in predicting survival, statistical significance was not observed for the differences between alteration of CD8^+^ T cell and some other indexes, implying that the need of improvement in predicting short-term survival with the changes of immune cells. Maybe the magnitude of effector T cell was more positively associated with long-term survival than short-term survival [36].

As the first study to compare the changes of immunocytes and to explore the prognostic power of these changes in patients with LAPC after IRE, our study was limited by the small size and retrospective nature. The immunocytes measured in this study did not represent all the components in the microenvironment of LAPC after IRE therapy. Moreover, maybe it is necessary to analyze the immune parameters withdrawn at the moment of progression. A longer follow-up period is also needed for the comparisons of effects of immunocytes on survival in LAPC patients and an external validation is also needed.

## 5. Conclusions

The present study showed the first picture of immunomodulatory of IRE in patients with LAPC. Alteration of CD8^+^ T cell was established as prognostic factor for OS and PFS and showed better prognostic value for survival prediction in LAPC patients after IRE therapy. The changes of CD8^+^ T between D3 and D7 after IRE could be used as a monitor factor of IRE treatment and a prognostic indicator of survival in LAPC patients after IRE therapy.

## Figures and Tables

**Table 1:** Characteristics of patients with LAPC undergoing IRE therapy.

Characteristics		Number	Percentage (%)
Patients		34	100
Age (years)	≤ 60	19	55.9
	> 60	15	44.1
Gender	Female	18	52.9
	Male	16	47.1
Tumor size (cm)	≤ 2	1	2.9
	2~4	19	55.9
	>4	14	41.2
Tumor grade	Well	2	5.9
	Moderate	19	55.9
	Poor	13	38.2
Tumor site	Head	17	50.0
	Body / Tail	17	50.0
WBC (*∗*109)	≤ 10	30	88.2
	> 10	4	11.8
HGB (g/L)	≤ 120	10	29.4
	> 120	24	70.6
PLT (*∗*109)	≤ 300	29	85.3
	> 300	5	14.7
ALT (U/L)	≤ 40	25	73.5
	> 40	9	26.5
AST (U/L)	≤ 40	28	82.4
	> 40	6	17.6
ALP (U/L)	≤ 100	18	52.9
	> 100	16	47.1
GGT (U/L)	≤ 45	18	52.9
	> 45	16	47.1
ALB (g/L)	≤ 40	3	8.8
	> 40	31	91.2
TBIL (umol/L)	≤ 20.5	26	76.5
	> 20.5	8	23.5
IBIL (umol/L)	≤ 15	30	88.2
	> 15	4	11.8
CRP (ng/L)	≤ 3	25	73.5
	> 3	9	26.5
CEA (ng/mL)	≤ 5	20	58.8
	> 5	14	41.2
CA19-9 (U/ml)	≤ 35	8	23.5
	> 35	26	76.5
NLR	≤ 1.47	9	26.5
	> 1.47	25	73.5
PLR	≤ 165.29	22	64.7
	> 165.29	12	35.3
PI	0	25	73.5
	1	9	26.5
mGPS	0	29	85.3
	1	4	11.8
	2	1	2.9

LAPC, locally advanced pancreatic cancer; IRE, irreversible electroporation; WBC, white blood cell count; PLT, platelet count; ALT, alanine transaminase; AST, aspartate aminotransferase; ALP, alkaline phosphatase; GGT, glutamyl transpeptidase; ALB, albumin; TBIL, total bilirubin; IBIL, indirect bilirubin; CRP, C-reactive protein; CEA, carcinoembryonic antigen; CA19-9, carbohydrate antigen 19-9; NLR, neutrophil-to-lymphocyte ratio; PLR, platelet-to-lymphocyte ratio; PI, prognostic index; mGPS, modified Glasgow Prognostic Score.

**Table 2:** Complications after IRE treatment in patients with LAPC.

Complications	Number
Hypotension	3
Hypokalemia	2
Fatigue	2
Vomiting	1
Diarrhea	2
Thrombosis	2
Ascites	1
Pain	3
Muscle weakness	1

Abbreviations as in Table 1

**Table 3:** Univariate and multivariate analyses of OS and PFS in patients.

Characteristic		OS						PFS					
		Univariate analysis			Multivariate analysis			Univariate analysis			Multivariate analysis		
		HR	95%CI	P	HR	95%CI	P	HR	95%CI	P	HR	95% CI	P
Age (years)	≤ 60 / > 60	0.753	0.163-3.377	0.711			NI	0.988	0.379-2.577	0.981			NI
Gender	Female / Male	8.535	0.994-72.563	0.055			NI	1.535	0.577-4.084	0.391			NI
Tumor size (cm)	>4 / 2~4 / ≤ 2	1.405	0.405-4.876	0.592			NI	1.019	0.412-2.522	0.967			NI
Tumor grade	Poor / Moderate / Well	2.754	0.668-11.354	0.161			NI	0.910	0.391-2.117	0.827			NI
Tumor site	Head / Body / Tail	3.778	0.743-19.204	0.109			NI	0.848	0.393-1.830	0.674			NI
WBC (*∗*10^9^)	≤ 10 / > 10	0.733	0.087-6.164	0.775			NI	1.019	0.412-2.522	0.967			NI
HGB (g/L)	≤ 120 / > 120	0.544	0.121-2.445	0.427			NI	1.202	0.377-4.280	0.777			NI
PLT (*∗*10^9^)	≤ 300 / > 300	1.588	0.289-8.721	0.595			NI	0.477	0.107-2.116	0.330			NI
ALT (U/L)	≤ 40 / > 40	0.995	0.191-5.181	0.995			NI	0.409	0.117-1.431	0.162			NI
AST (U/L)	≤ 40 / > 40	0.033	0.000-67.662	0.382			NI	0.596	0.168-2.112	0.423			NI
ALP (U/L)	≤ 100 / > 100	0.832	0.185-3.742	0.811			NI	0.522	0.193-1.417	0.202			NI
GGT (U/L)	≤ 45 / > 45	1.404	0.310-6.365	0.660			NI	0.418	0.136-1.289	0.129			NI
ALB (g/L)	≤ 40 / > 40	0.572	0.066-4.939	0.611			NI	0.924	0.206-4.149	0.918			NI
TBIL (umol/L)	≤ 20.5 / > 20.5	0.033	0.000-67.662	0.382			NI	0.596	0.168-2.112	0.423			NI
IBIL (umol/L)	≤ 15 / > 15	0.040	0.000-95.774	0.530			NI	1.148	0.328-4.022	0.829			NI
CRP (ng/L)	> 3 / ≤ 3	8.328	1.443-48.063	0.018	2.458	0.048-125.03	0.654	1.024	0.275-3.817	0.972			NI
CEA (ng/mL)	≤ 5 / > 5	0.999	0.193-5.161	0.999			NI	2.106	0.805-5.512	0.129			NI
CA19-9 (U/ml)	≤ 35 / > 35	0.865	0.165-4.554	0.868			NI	2.635	0.573-11.523	0.178			NI
CD4^+^ T cell variation	Elevated/Non-elevated	0.214	0.041-1.127	0.069			NI	0.920	0.343-2.470	0.869			NI
CD8^+^ T cell variation	Elevated/Non-elevated	0.056	0.006-0.490	0.009	0.039	0.002-0.780	0.034	0.354	0.122-1.026	0.046	0.418	0.138-0.954	0.049
NK cell variation	Elevated/Non-elevated	0.144	0.025-0.816	0.029	0.184	0.005-6.745	0.357	0.817	0.293-2.277	0.700			NI
Treg cell variation	Decreased/Non-decreased	0.165	0.033-0.820	0.028	1.056	0.026-43.365	0.977	0.452	0.166-1.228	0.119			NI
IL-2 variation	Elevated/Non-elevated	0.393	0.075-2.067	0.270			NI	0.408	0.116-1.438	0.163			NI
IL-6 variation	Decreased/Non-decreased	0.851	0.188-3.841	0.834			NI	0.645	0.190-2.187	0.482			NI
IL-10 variation	Decreased/Non-decreased	1.271	0.281-5.757	0.756			NI	1.262	0.396-4.020	0.694			NI
C3 variation	Elevated/Non-elevated	1.172	0.260-5.292	0.837			NI	1.386	0.419-4.584	0.592			NI
C4 variation	Elevated/Non-elevated	1.201	0.241-5.974	0.823			NI	1.458	0.434-4.893	0.542			NI
IgG variation	Elevated/Non-elevated	4.012	0.411-39.156	0.232			NI	1.459	0.237-8.985	0.684			NI
NLR	> 1.47 / ≤ 1.47	5.967	0.680-52.400	0.107			NI	3.185	1.068-13.621	0.039	3.425	1.002 - 12.616	0.046
PLR	> 165.29 / ≤ 165.29	3.752	0.738-19.073	0.111			NI	1.517	0.552-4.171	0.419			NI
PI	2 / 1 / 0	3.864	0.861-17.333	0.078			NI	1.292	0.439-3.804	0.642			NI
mGPS	2 / 1 / 0	3.264	1.266-8.413	0.014	4.285	0.355-51.673	0.252	0.921	0.284-2.987	0.891			NI

OS, overall survival; PFS, progression-free survival; NI, not included; Other abbreviations as in Table 1

**Table 4:** Comparison of the values of AUC.

	CD8^+^ T cell variation	CD4^+^ T cell variation	NK cell variation	Treg cell variation	NLR	PLR	PI	mGPS
OS	0.816	0.639	0.608	0.460	0.722	0.534	0.601	0.654
PFS	0.773	0.477	0.598	0.480	0.691	0.503	0.674	0.540

Abbreviations as in Table 3

**Table 5:** Comparison of the values of C-indexes.

		CD8+ T cell variation	CD4+ T cell variation	NK cell variation	Treg cell variation	NLR	PLR	PI	mGPS
OS	value	0.816 (0.711-0.921)	0.698 (0.554-0.842)	0.755 (0.619-0.891)	0.764 (0.636-0.892)	0.632 (0.446-0.818)	0.670 (0.484-0.856)	0.675 (0.478-0.872)	0.693 (0.525-0.861)
CD8+ T cell variation	P value		0.008	0.162	0.210	0.011	0.007	0.068	0.103
CD4+ T cell variation	P value	0.008		0.188	0.164	0.275	0.365	0.409	0.482
NK cell variation	P value	0.162	0.188		0.452	0.112	0.113	0.235	0.281
Treg cell variation	P value	0.210	0.164	0.452		0.081	0.086	0.212	0.252
NLR	P value	0.011	0.275	0.112	0.081		0.343	0.349	0.265
PLR	P value	0.007	0.365	0.113	0.086	0.343		0.485	0.415
PI	P value	0.068	0.409	0.235	0.212	0.349	0.485		0.425
mGPS	P value	0.103	0.482	0.281	0.252	0.265	0.415	0.425	

PFS	value	0.639 (0.523-0.755)	0.520 (0.381-0.659)	0.541 (0.407-0.675)	0.614 (0.485-0.743)	0.661 (0.579-0.743)	0.563 (0.436-0.690)	0.505 (0.382-0.628)	0.475 (0.365-0.585)
CD8+ T cell variation	P value		0.081	0.084	0.368	0.376	0.116	0.020	0.006
CD4+ T cell variation	P value	0.081		0.407	0.148	0.013	0.298	0.423	0.260
NK cell variation	P value	0.084	0.407		0.207	0.023	0.399	0.309	0.200
Treg cell variation	P value	0.368	0.148	0.207		0.264	0.219	0.083	0.014
NLR	P value	0.376	0.013	0.023	0.264		0.055	0.002	0.001
PLR	P value	0.116	0.298	0.399	0.219	0.055		0.257	0.078
PI	P value	0.020	0.423	0.309	0.083	0.002	0.257		0.327
mGPS	P value	0.006	0.260	0.200	0.014	0.001	0.078	0.327	

Abbreviations as in Table 3

## Data Availability

The datasets from SYSUCC dataset are available from the corresponding author upon reasonable request.
